# Factors associated with psychiatric consultation history and methods of suicide in individuals who died by suicide in Osaka City: A retrospective study

**DOI:** 10.1002/pcn5.70092

**Published:** 2025-04-07

**Authors:** Ryu Murakami, Daigo Morioka, Kenko Fukui, Atsushi Hiraide, Hisanaga Kuroki

**Affiliations:** ^1^ Meiji University of Integrative Medicine, School of Health Science and Medical Care, Faculty of Emergency Medical Science Nantan City Japan; ^2^ Chiba Institute of Science, Graduate School of Risk and Crisis Management Choshi City Japan; ^3^ Osaka Prefectural Medical Examiner's Office Osaka City Japan

**Keywords:** suicide, psychiatric consultations, Cochran–Armitage trend, postmortem, mental health

## Abstract

**Aim:**

This retrospective study investigated the relationship among psychiatric consultation history, method of suicide, and the background information of individuals who died by suicide, based on postmortem information obtained from the Osaka Prefectural Medical Examiner's Office.

**Methods:**

We analyzed the data of 343 cases of suicide that occurred in Osaka City in 2017, focusing on factors associated with a history of psychiatric consultations. The Cochran–Armitage trend test was used to evaluate whether there was a significant linear trend in the distribution of case counts across 10‐year age groups. Univariate and multivariate logistic regression analyses were used to identify the factors associated with a history of psychiatric consultations prior to death.

**Results:**

The results revealed that female sex, history of suicide attempts, and choosing jumping as the suicide method compared to choosing hanging were the significant factors associated with a history of psychiatric consultations prior to death. Factors associated with not having a history of psychiatric consultations prior to death were being employed (compared to being unemployed), being a student (compared to being unemployed), and being older (compared to being younger). The Cochran–Armitage trend test revealed no significant linear trend in the distribution of case counts.

**Conclusion:**

The study highlights the need for targeted mental health interventions for specific demographic groups, as well as further research on the impact of mental health conditions and age‐related factors on suicide methods. These results may contribute to a deeper understanding of the risk factors for suicide and help in improving suicide prevention strategies.

## Graphical abstract

This retrospective study investigated the relationship among psychiatric consultation history, method of suicide, and the background information of individuals who died by suicide, based on postmortem information obtained from the Osaka Prefectural Medical Examiner's Office. We analyzed the data of 343 cases of suicide that occurred in Osaka City in 2017, focusing on factors associated with a history of psychiatric consultations. The results revealed that female sex, history of suicide attempts, and choosing jumping as the suicide method compared to choosing hanging were the significant factors associated with a history of psychiatric consultations prior to death. Factors associated with not having a history of psychiatric consultations prior to death were being employed (compared to being unemployed), being a student (compared to being unemployed), and being older (compared to being younger).

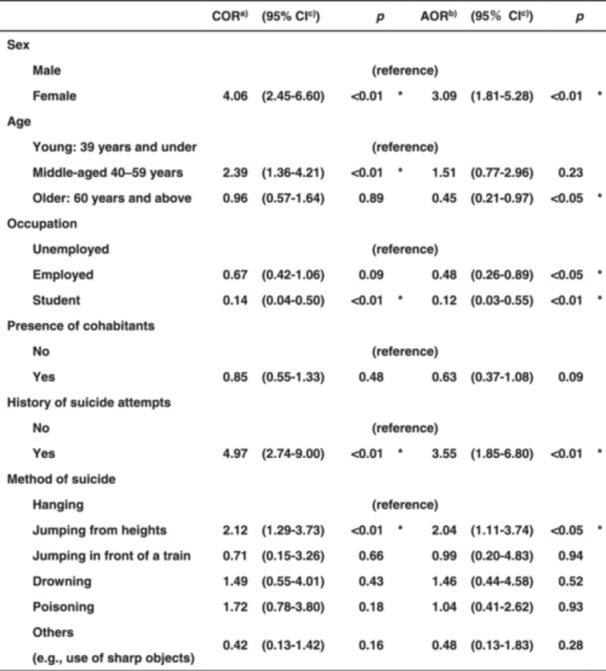

## INTRODUCTION

Suicide is a significant public health issue that must be addressed globally. According to a World Health Organization (WHO) report, suicide occurs not only in high‐income countries but also in low‐ and middle‐income countries, and it is among the leading causes of death worldwide among young people aged 15–29 years.^1^, ^2^ WHO also emphasizes the importance of surveillance on suicide and self‐harm for effective suicide prevention strategies.^3^

Suicidal behavior has been suggested by studies worldwide to be associated with mental disorders in various populations.^4^, ^5^, ^6^ Hence, understanding the relationship between suicidal behavior and mental disorders is a crucial factor in suicide prevention. Reportedly, suicide attempters with higher medical severity exhibited more severe psychological impairments, such as depression, agitation, and self‐loathing.^7^ The ultimate and most tragic outcome of suicidal behavior is death by suicide. Cases resulting in death from suicidal acts are associated with various suicide methods.^8^ Investigating these methods from various perspectives and exploring the background of these individuals is vital for deepening our understanding of suicidal behavior.

In many countries, suicide is classified as an “unnatural death.” For example, in Australia, the Coroners Court is responsible for investigating suicide cases in each state, and information on external cause deaths, including suicides, is collected through the National Coronial Information System.^4^, ^9^ Also in Japan, suicides are classified as “unnatural deaths” and are investigated by the police. However, there is no extensive forensic database, and comprehensive studies using forensic data to investigate suicide cases, focusing on variables related to psychiatry and the methods of suicide, remain scarce in Japan.

The objective of this study was to elucidate the relationship among a history of psychiatric consultations prior to death, methods of suicide, and the personal background information of individuals who died by suicide.

## METHODS

### Research design

This retrospective, observational study analyzed the information of individuals who died by suicide, which was derived from documents submitted by the police to the medical examiner when requesting a postmortem examination (hereafter referred to as ‘target documents’). These documents contain detailed information on cases of unnatural deaths that were identified as suicides through police investigations, including the deceased's pension status, occupation, psychiatric history, method of suicide, history of previous suicide attempts, and presence of cohabitants, which were obtained through inquiries made to various institutions such as hospitals and administrative agencies. This study focused on individuals who died by suicide and analyzed the relationship between mental disorders, which are known risk factors for suicide, and the methods of suicide, as well as the personal background information of individuals who died by suicide. The data were obtained from documents managed by the Osaka Prefectural Medical Examiner's Office, which oversees Osaka City, the second most densely populated city in Japan after Tokyo among those cities with a medical examiner system in place. The study was conducted with the approval of the Human Ethics Review Committee of Meiji University of Integrative Medicine (Approval No.: 2022‐039). For accessing the target documents in this study, approval was obtained from the Osaka Prefectural Medical Examiner's Office.

### Content of the target documents

The target documents included the following key information: (1) date and time of discovery of death by suicide, (2) sex, (3) age (recorded as an exact number), (4) pension status, (5) status of receiving health and welfare services, (6) occupation, (7) presence of cohabitants, (8) history of psychiatric consultations, (9) method of suicide, and (10) a remarks section where the police investigator responsible for the case can freely give an overview of the unnatural death. Except for the age and remarks sections, the other items were provided in a standardized checklist format. We organized these items into a database using Excel (Microsoft Corporation).

### Participants

This study investigated the anonymized data of 569 individuals who died by suicide in Osaka City during the study period from January 1, 2017 to December 31, 2017, as recorded in the target documents. All the data on suicides in this study pertained to cases that resulted in death. Consequently, among cases where the body was discovered in a highly decomposed state, there were cases where the personal information was unknown or cases where, despite the identification of the deceased, the immediate circumstances leading to the death were not fully understood at the time the police submitted the documents to the medical examiner (In this case, the target documents will be marked “still under investigation” to clarify that this is an item for which detailed information is not yet available). Therefore, cases with missing values for any of the evaluation items or explanatory variables were excluded from the study.

### Statistical analysis

To summarize the characteristics of the participants, categorical variables were expressed as counts and percentages. Univariate and multivariate logistic regression analyses were used to identify the factors associated with a history of psychiatric consultations prior to death and the crude odds ratio (COR), adjusted odds ratio (AOR), and 95% confidence intervals (CIs) were estimated.

The primary outcome measure of this study was a history of psychiatric consultations (Yes/No).

The explanatory variables were as follows:

1. Sex (a, male; b, female)
2. Age (a, young 39 years and under; b, middle‐aged 40–59 years; c, older 60 years and above)
3. Occupation (a, unemployed; b, employed; c, student)
4. Presence of cohabitants (yes/no)
5. History of suicide attempts (yes/no)
6. Method of suicide (a, hanging; b, jumping from heights; c, jumping in front of a train; d, drowning; e, poisoning; f, others, e.g. use of sharp objects)

Suicide occurs as a result of complex interactions among various factors. Based on this premise, this study analyzed data derived from information about individuals who died by suicide, which has been organized into pre‐categorized document layouts. Therefore, the cases were analyzed by considering them as part of larger groups to some extent. We divided the age groups into three categories to perform logistic regression analysis. The age classifications defined by the Japanese government vary depending on the policy and are not uniformly defined by the Cabinet Office. Therefore, in this study, the age group up to 39 years, for which suicide was the leading cause of death in 2017, was classified as the young age group.^10^ Following the age classification for the young age group, it was necessary to define the middle‐aged and older‐aged groups. In Japan, many companies commonly set the retirement age at 60 years or older; hence, individuals aged 60 years and above were classified as the older‐aged group. These classifications were also based on previous studies.^11^, ^12^ Prior to conducting the logistic regression analysis, participants were initially classified into 10‐year age groups to describe trends in the increase or decrease in the number of suicides within finer age distributions. The Cochran–Armitage trend test was then used to evaluate whether there was a significant linear trend in the distribution of case numbers across these age groups.

The “Pension status” variable in the target documents is generally influenced by the pension eligibility age defined by the Japanese government; hence, younger individuals are automatically classified as non‐recipients, which may introduce bias. The “Status of receiving health and welfare services” variable in the target documents whether individuals were receiving such services or not. However, Japan does not operate under a single unified health and welfare service system; instead, a variety of services are provided across different age groups. Therefore, it was deemed challenging to track which specific services were received in individual cases, and it was anticipated that this would complicate the interpretation of multivariate analysis. The authors (R.M., D.M., K.F., A.H., and H.K.) thus reached a consensus to exclude these two variables from the logistic regression analysis.

Statistical significance was set at *α* = 0.05 (two‐sided). All statistical analyses in this study were performed using IBM SPSS Statistics Ver.29.0.0.0 (IBM Corporation).

## RESULTS

### Background information of the suicide cases

**Figure 1:**
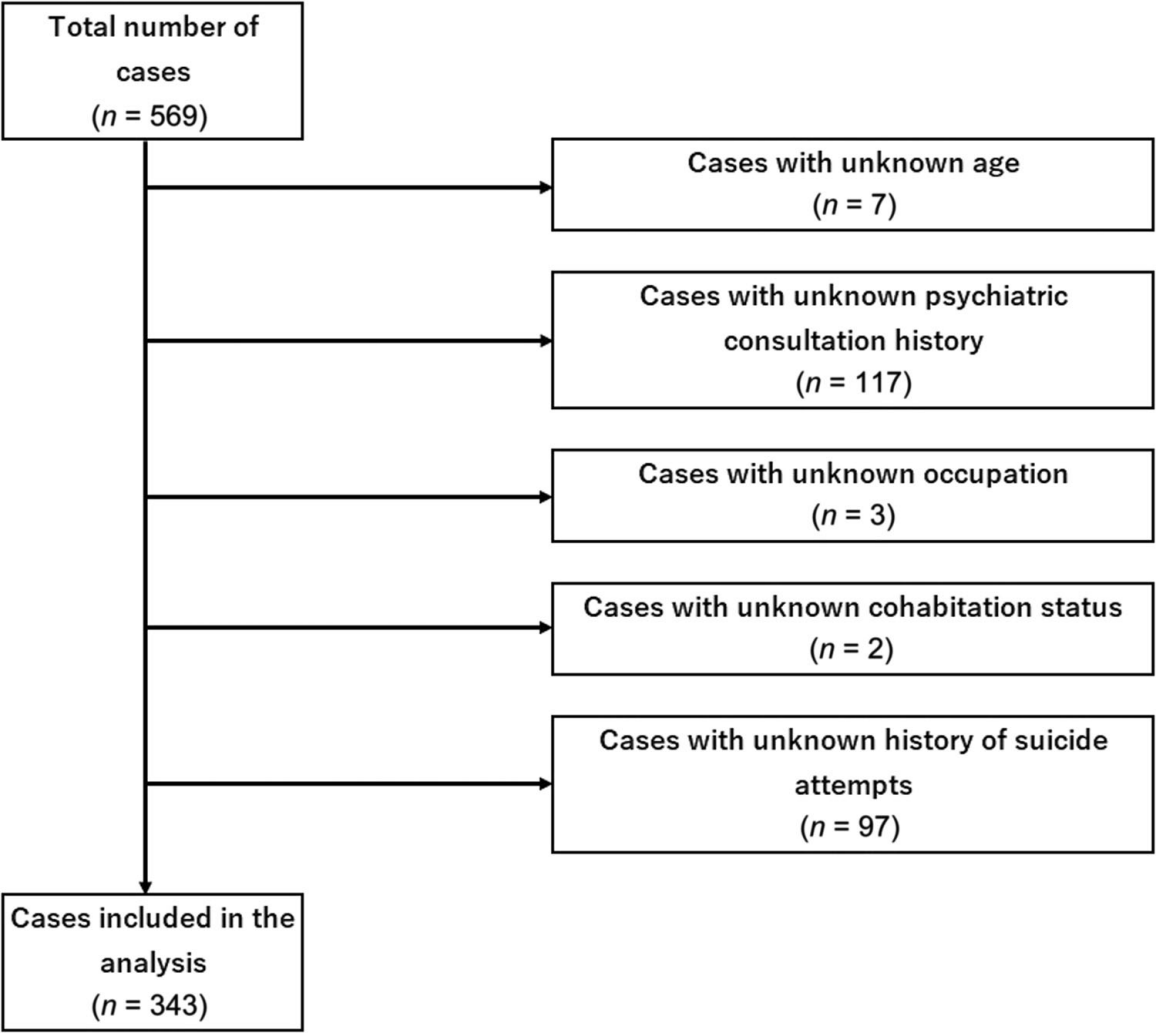
Exclusion criteria. Of the 569 recorded suicide cases during the study period, 343 cases were analyzed after excluding those with missing data.

Of the 569 recorded suicide cases during the study period, 343 cases were analyzed after excluding those with missing data (Figure 1). The background characteristics of the included cases are shown in Table 1. In terms of sex distribution, there were 210 men (61.2%) and 133 women (38.8%). When the age of the cases was categorized into 10‐year age groups, the Cochran–Armitage trend test revealed no significant linear trend in the distribution of case counts (P = 0.75). Regarding occupation, unemployed individuals accounted for the largest proportion, with 213 cases (62.1%). In terms of cohabitation status, 212 cases (61.8%) involved individuals living with others. Regarding the history of suicide attempts, 253 cases (73.8%) had no history of suicide attempts. Additionally, 196 cases (57.1%) had a history of psychiatric consultations. The most common method of suicide was hanging, with 181 cases (52.8%), followed by jumping from heights, with 93 cases (27.1%).

**Table 1:** Classification of individuals who died by suicide based on the presence or absence of psychiatric consultation history.

Variables	Total number	History of psychiatric consultations	
		Yes	No
*n* (%)	343 (100)	196 (57.1)	147 (42.9)
Sex			
Male	210 (61.2)	94 (48.0)	116 (78.9)
Female	133 (38.8)	102 (52.0)	31 (21.1)
Age groups			
Young: under 39 years	95 (27.7)	48 (24.5)	47 (32.0)
Middle‐aged: 40–59 years	117 (34.1)	83 (42.3)	34 (23.1)
Older: 60 years and above	131 (38.2)	65 (33.2)	66 (44.9)
Sub‐age groups*			
10–19 years	13 (3.8)	1 (0.5)	12 (8.2)
20–29 years	40 (11.7)	24 (12.2)	16 (10.9)
30–39 years	42 (12.2)	23 (11.7)	19 (12.9)
40–49 years	57 (16.6)	40 (20.4)	17 (11.6)
50–59 years	60 (17.5)	43 (21.9)	17 (11.6)
60–69 years	48 (14.0)	26 (13.3)	22 (15.0)
70–79 years	53 (15.5)	25 (12.8)	28 (19.0)
80–89 years	26 (7.6)	13 (6.6)	13 (8.8)
90–99 years	4 (1.2)	1 (0.5)	3 (2.0)
Occupation			
Unemployed	213 (62.1)	133 (67.9)	80 (54.4)
Employed	114 (33.2)	60 (30.6)	54 (36.7)
Student	16 (4.7)	3 (1.5)	13 (8.8)
Presence of cohabitants			
No	131 (38.2)	78 (39.8)	53 (36.1)
Yes	212 (61.8)	118 (60.2)	94 (63.9)
History of suicide attempts			
No	253 (73.8)	122 (62.2)	131 (89.1)
Yes	90 (26.2)	74 (37.8)	16 (10.9)
Method of suicide			
Hanging	181 (52.8)	93 (47.4)	88 (59.9)
Jumping from heights	93 (27.1)	65 (33.2)	28 (19.0)
Jumping in front of a train	7 (2.0)	3 (1.5)	4 (2.7)
Drowning	18 (5.2)	11 (5.6)	7 (4.8)
Poisoning	31 (9.0)	20 (10.2)	11 (7.5)
Others (e.g., use of sharp objects)	13 (3.8)	4 (2.0)	9 (6.1)

* Cochran–Armitage trend test: *p* = 0.75.

### Factors associated with psychiatric consultations among individuals who died by suicide

The results of the multivariate logistic regression analysis, using the presence or absence of a history of psychiatric consultations as the dependent variable, are presented in Table 2. After adjusting for each explanatory variable, the factors that showed a significant association with a history of psychiatric consultations were as follows: sex (female) (AOR 3.09, 95% CI 1.81–5.28), age (older group compared to the young group) (AOR 0.45, 95% CI 0.21–0.97), occupation (employed compared to unemployed) (AOR 0.48, 95% CI 0.26–0.89), occupation (student compared to unemployed) (AOR 0.12, 95% CI 0.03–0.55), history of suicide attempts (AOR 3.55, 95% CI 1.85–6.80), and method of suicide (jumping compared to hanging) (AOR 2.04, 95% CI 1.11–3.74).

**Table 2:** Analysis of factors associated with psychiatric consultation history among individuals who died by suicide.

	COR	(95% CI)	*p*		AOR	(95％ CI)	*p*	
Sex								
Male	(reference)							
Female	4.06	(2.45–6.60)	<0.01	[*]	3.09	(1.81–5.28)	<0.01	[*]
Age								
Young: 39 years and under	(reference)							
Middle‐aged 40–59 years	2.39	(1.36–4.21)	<0.01	[*]	1.51	(0.77–2.96)	0.23	
Older: 60 years and above	0.96	(0.57–1.64)	0.89		0.45	(0.21–0.97)	<0.05	[*]
Occupation								
Unemployed	(reference)							
Employed	0.67	(0.42–1.06)	0.09		0.48	(0.26–0.89)	<0.05	[*]
Student	0.14	(0.04–0.50)	<0.01	[*]	0.12	(0.03–0.55)	<0.01	[*]
Presence of cohabitants								
No	(reference)							
Yes	0.85	(0.55–1.33)	0.48		0.63	(0.37–1.08)	0.09	
History of suicide attempts								
No	(reference)							
Yes	4.97	(2.74–9.00)	<0.01	[*]	3.55	(1.85–6.80)	<0.01	[*]
Method of suicide								
Hanging	(reference)							
Jumping from heights	2.12	(1.29–3.73)	<0.01	[*]	2.04	(1.11–3.74)	<0.05	[*]
Jumping in front of a train	0.71	(0.15–3.26)	0.66		0.99	(0.20–4.83)	0.94	
Drowning	1.49	(0.55–4.01)	0.43		1.46	(0.44–4.58)	0.52	
Poisoning	1.72	(0.78–3.80)	0.18		1.04	(0.41–2.62)	0.93	
Others	0.42	(0.13–1.42)	0.16		0.48	(0.13–1.83)	0.28	
(e.g., use of sharp objects)								

*Note*: No multicollinearity was observed for any of the variables (VIF < 9). The area under the curve was 0.78.

Abbreviations: AOR, adjusted odds ratio; CI, confidence interval; COR, crude odds ratio.

* *p* < 0.05.

## DISCUSSION

### Analysis of factors associated with psychiatric consultation history among individuals who died by suicide

The results of the multivariate logistic regression analysis (Table 2) revealed that being female was a significant factor associated with a history of psychiatric consultations prior to death among those who died by suicide. This result suggests that sex has some association with the history of psychiatric consultations. Previous studies on healthcare‐seeking behavior ^13^, ^14^ have reported that women are more likely than men to visit primary care providers for both physical and mental health issues. Additionally, some reports suggest that masculinity is associated with a lower likelihood of seeking mental health support.^15^, ^16^, ^17^ Therefore, the relationship between sex and mental health support should be carefully examined in future studies.

The results of this study suggest that while it is important to provide proactive support to women visiting primary care, identifying and screening men who do not visit primary care may also be a critical challenge for future suicide prevention policies.

The results of the Cochran–Armitage trend test (Table 1) did not reveal a statistically significant linear trend, implying that changes in age do not necessarily exhibit a linear increase or decrease in relation to the presence or absence of psychiatric consultations. This suggests that age categorization of persons who died by suicide should not necessarily be based on 10‐year age groups but rather on stratification that considers their social background factors. Therefore, the classification method by age group requires further investigation.

The middle‐aged group compared to the younger group did not demonstrate a significant association with a psychiatric consultation history. However, the older group was associated with not seeking psychiatric consultations compared to the younger group. Reportedly, in individuals who died by suicide without receiving mental health services, non‐utilization of mental health services was associated with both younger and older individuals.^18^ Alternatively, a lower perceived need for mental health treatment may be a contributing factor.^19^ In the older‐aged group, retirement from work is likely, which may lead to fewer opportunities for health check‐ups or concern for their physical condition compared to others in work‐related settings. Psychiatric consultations may be influenced by the prevalence of age‐dependent diseases, such as dementia. However, in our current study, we did not analyze individual psychiatric disorder diagnoses in each case. We therefore believe it is necessary to analyze the specific psychiatric disorders diagnosed in cases with a history of psychiatric consultation. As another hypothesis, we consider that barriers to accessing psychiatric services and information about mental illnesses, the unfamiliarity of older adults with information technology,^20^ and physical barriers such as the lack of means to travel to service areas may contribute to the low rates of psychiatric consultations among older adults. Moving forward, further investigation into the actual utilization of psychiatric services by older adults is necessary.

The results of this study indicate that among those who died by suicide, workers and students, compared to unemployed individuals, are for some reason not seeking psychiatric consultations. Being employed was identified as a factor associated with not having a history of psychiatric consultation. This result aligns with our previous findings.^21^ Stigma related to mental health may contribute to the reluctance to seek mental health support in various ways. Particularly among young individuals and the working‐age population, barriers to acknowledging mental health issues and seeking help have been discussed in different contexts.^4^, ^22^, ^23^ The results of this study suggest that these barriers may have contributed to the observed outcomes by influencing psychiatric consultation for these people. Additionally, we propose that promoting psychiatric education targeted at worker and student populations could be an effective strategy for suicide prevention.

A history of suicide attempts was, in line with the results of previous studies, a factor associated with a history of psychiatric consultations.^12^, ^21^ In cases of non‐fatal suicidal behavior, it is possible that third parties who witnessed the behavior called emergency services or that the worsening symptoms of a mental disorder led to the non‐fatal suicidal behavior. However, it was difficult to determine from the target documents whether the suicide attempt led to the psychiatric consultation or if the deterioration of the mental disorder preceded the suicidal behavior, therefore additional studies are needed to explore this aspect.

In terms of the methods of suicide, jumping from heights, compared to hanging, was associated with a history of psychiatric consultations. A history of psychiatric consultations suggests that the individuals who died by suicide may have been suffering from a mental disorder before their death. Thus, this finding implies a potential relationship among the chosen method of suicide, the presence of a mental disorder, and the act of seeking psychiatric care. Moreover, sex differences in the choice of suicide methods may explain the sex differences in suicide mortality rates.^24^, ^25^ While this study did not examine the relationship between sex and methods of suicide, the sex ratio of the study participants (i.e., the sex differences in suicide deaths) indicated a higher proportion of male deaths, suggesting a possible interaction among suicide methods, psychiatric consultation history, and sex.

It is worth noting that the relationship between psychiatric consultation history and methods of suicide in this study was based on analysis of the forensic data of individuals who died by suicide in Japan. We believe that the findings of this study, combined with the existing knowledge on suicide, could help in strengthening suicide prevention measures.

### Limitations of the study

This study has some limitations. It was based on medical information obtained through police inquiries to medical institutions and other sources at the time the target documents reached the medical examiner, therefore it is important to note that there may be information that was not yet known at the time the documents were sent to the medical examiner. Additionally, as this study is based on document records, there may be unreported suicide attempts (including cases where old injuries that could reasonably indicate a history of suicide attempts were not observed) that occurred in environments without witnesses, which may not have been captured in the study data. Since this study was conducted in a single region, the findings may not be immediately generalizable to all suicide cases across all regions. It is also important to acknowledge the potential effect of confounding factors that were not included in the dataset used for this study.

Moreover, this study focused only on individuals who died by suicide and did not consider cases where suicide was not completed. It was also not possible to track the specific reasons for which the deceased sought psychiatric care prior to their death. Finally, the study could not determine the temporal relationship between psychiatric consultations and suicide attempts, making it difficult to ascertain which occurred first.

## CONCLUSIONS

In conclusion, this study investigated the relationship among psychiatric consultation history, methods of suicide, and the personal background information of individuals who died by suicide based on postmortem information. The factors associated with having a history of psychiatric consultations prior to death were being female, having a history of suicide attempts, and choosing jumping as the suicide method compared to hanging. Factors associated with not having a history of psychiatric consultations prior to death were being a student (compared to being unemployed) and being older (compared to being younger).

Future studies should aim to conduct more comprehensive analyses of the mental disorders affecting the individuals who died by suicide, the chosen methods of suicide, and the background information of individuals who died by suicide across different age groups. Understanding these factors may provide new insights and strengthen suicide prevention strategies.

## AUTHOR CONTRIBUTIONS

Ryu Murakami contributed to the writing of the manuscript, data collection, analysis, and overall supervision of the study. Daigo Morioka contributed to data collection, analysis, and verification of the content structure. Kenko Fukui was involved in data analysis and interpretation, as well as the verification of the content structure. Atsushi Hiraide provided advice on the manuscript content and guidance on statistical analysis. Hisanaga Kuroki contributed to data collection, provided advice on the manuscript content, and offered guidance on statistical analysis.

## CONFLICT OF INTEREST STATEMENT

The authors declare no conflicts of interest.

## ETHICS APPROVAL STATEMENT

The study was conducted with the approval of the Human Ethics Review Committee of Meiji University of Integrative Medicine (Approval No.: 2022‐039). For accessing the target documents in this study, approval was obtained from the Osaka Prefectural Medical Examiner's Office.

## PATIENT CONSENT STATEMENT

This study was conducted in accordance with an opt‐out procedure. The opt‐out document, prepared in compliance with Japan's ethical guidelines, allows the families of the deceased to freely request exclusion from the study if they wished. This document is available on the website of the research institution to which the first author belongs, and printed versions are posted and available at designated locations within the author's affiliated institution.

## CLINICAL TRIAL REGISTRATION

N/A.

## Data Availability

The data used in this study cannot be publicly shared due to ethical considerations, personal information protection, and restrictions on non‐public data. The information on individuals who died by suicide used in this study is based on documents stored at the Osaka Prefectural Medical Examiner's Office, therefore this information is not publicly available. To conduct this study, materials were processed to ensure that individuals could not be identified. The statistical analyses in this study were conducted on information terminals that were disconnected from the internet and external networks. The data used were stored on encrypted storage media and strictly managed within the research institution to which the first author is affiliated.
